# Supplementary material for: Attention dynamics on the Chinese social media Sina Weibo during the COVID-19 pandemic
Source: EPJ Data Sci. 2021 Feb 3;10(1):8. doi: 10.1140/epjds/s13688-021-00263-0 (PMC7856455; doi:10.1140/epjds/s13688-021-00263-0)
Supplement: Supplementary file 1 — Supplementary information (PDF 2.3 MB) [file 13688_2021_263_MOESM1_ESM.pdf]

SUPPLEMENTARY INFORMATION

Attention dynamics on the Chinese social media Sina Weibo during the COVID-19 pandemic

Hao Cui and János Kertész*

*Correspondence: kerteszj@ceu.edu
Department of Network and Data Science, Central European University, Quellenstrasse 51, A-1100 Vienna, Austria

SI1 Twitter trending COVID-topics in the United States

Sina Weibo is the largest microblogging site in China, where Twitter, the worldwide most popular service of this kind does not operate. It is a natural idea to try to compare our observations made on Sina Weibo with Twitter attention dynamics. Unfortunately, there is no comparable statistics on Twitter to the HSL. Instead, Twitter has the service to inform about most retweeted hashtags during the last 24 hours updated on the minute basis and broken down to countries [1]. We have chosen to study the US tweets.

Categorization of tweets has been widely investigated [2, 3], including recent attempts to analyze the impact of COVID-related topics [4] on Twitter by analyzing the sentiments to 10 words related to COVID. Twitter even created a "COVID-19 stream" [5] to promote this type of research. In spite of these, a direct comparison of our results on Sina Weibo with Twitter is hindered by a number of factors, including the different characters of the listings, the different roles hashtags play in these services and the differences due to the scripts. Nevertheless, we tried to capture at least the overall trends (see Fig. SI1).

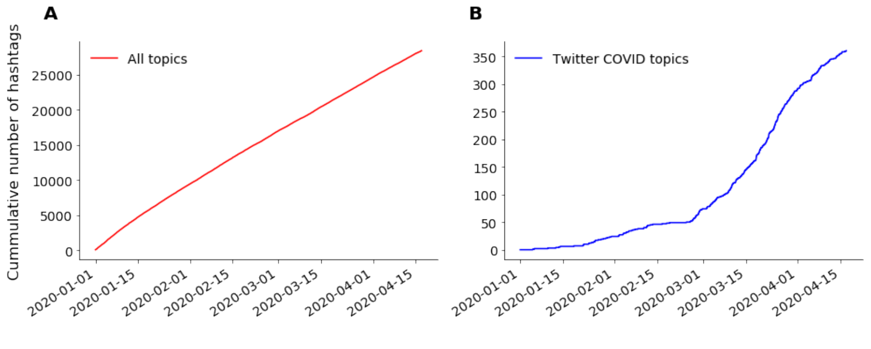

Figure SI1 Overview of the cumulative number of topics during the observation period on Twitter trending list in the United States from January 1, 2020 to April 16, 2020. (A) Cumulative growth of all topics. (B) Cumulative growth of COVID-related topics.

Fig. SI1 (A) shows the cumulative number of all the Twitter trending topics in the United States is almost perfectly linear. As Fig. SI1 (B) shows, the COVID-topics on Twitter trending list first grows very slowly at the beginning phase, and then starts to increase dramatically from late February 2020. The rate of COVID-related topics is, however, much smaller in the Twitter list than on that of the Sina Weibo.

SI2 Significance of correlations

To understand how the categories of time series of daily new hashtags move together and whether there are blocks of categories that co-move, we presented the correlation matrices plot between the ten time series in the three periods after the outbreak. In order to get information about the significance of the correlations we apply a null model, which is created by shuffling the times of the individual values, thus smearing out the correlations. Due to the finiteness of the time series, there will be non-zero background noise level denoted by Z in the null model, defining the background to which measured real correlations can be compared. Z is calculated by correlating 500 shuffled time series for each of the 10 categories. We observed that all the pairs have similar standard deviations between around 0.16 to 0.2. We take a uniform value $Z = 0.2$.

In Fig. SI2 we show correlations where only those C_{ij} correlation matrix elements are presented for which $Z < |C_{ij}|$. The figure shows the different Mainland China topical categories and their thresholded correlations in the three pandemic phases. In Fig. SI2 (B) most of the correlations are beyond the threshold, while in Fig. SI2 (D) very few are beyond the threshold. In Fig. SI2 (F), though some values at the upper left and lower right corners are beyond the threshold, they are much weaker than in Fig. SI2 (B).

SI3 Categorized Sina Weibo hashtags and properties

We showed in the main paper Fig. 4 that the gap between the top 15 ranks and the rest of the ranks in the rank diversity plot after the outbreak is caused by the COVID-hashtags. In order to further understand the properties of COVID-hashtags and how they influenced the HSL hashtag dynamics, we compared the highest rank and duration distribution of different COVID-categories with the non-COVID hashtags before and after the outbreak.

Fig. SI3 shows a detailed comparison of the highest rank and duration of the categorized Mainland China COVID-hashtags on Weibo Hot Search List (HSL), before and after the COVID-19 outbreak. As Fig. SI3 (A) shows, most of the categories have a median of highest rank close to 15. Science category and Bad News category are generally higher ranked than other categories. The median highest rank of the non-COVID hashtags after the outbreak is the same with that of the hashtags before the outbreak (rank 19), while the median highest rank of the COVID-hashtags is higher than both (rank 16). Fig. SI3 (B) shows the lifetime duration of the different categories. The median duration of most of the categories is less than 3.5 hours. Science category has the highest duration among all categories. Non-COVID hashtags after the outbreak (3.95 hours) and hashtags before the outbreak (3.80 hours) have similar duration distributions. The COVID-hashtags generally have shorter duration (3.21 hours) than non-COVID hashtags.

SI4 Hashtag rank trajectory examples

In the main paper, we have seen strange drops in the rank diversity plot at the ranks 29 and 34 after the outbreak, this implies that the number of unique hashtags occurred at these ranks in a given time interval is smaller than usual, so that there should be hashtags staying there for unusually long time. Here we present examples

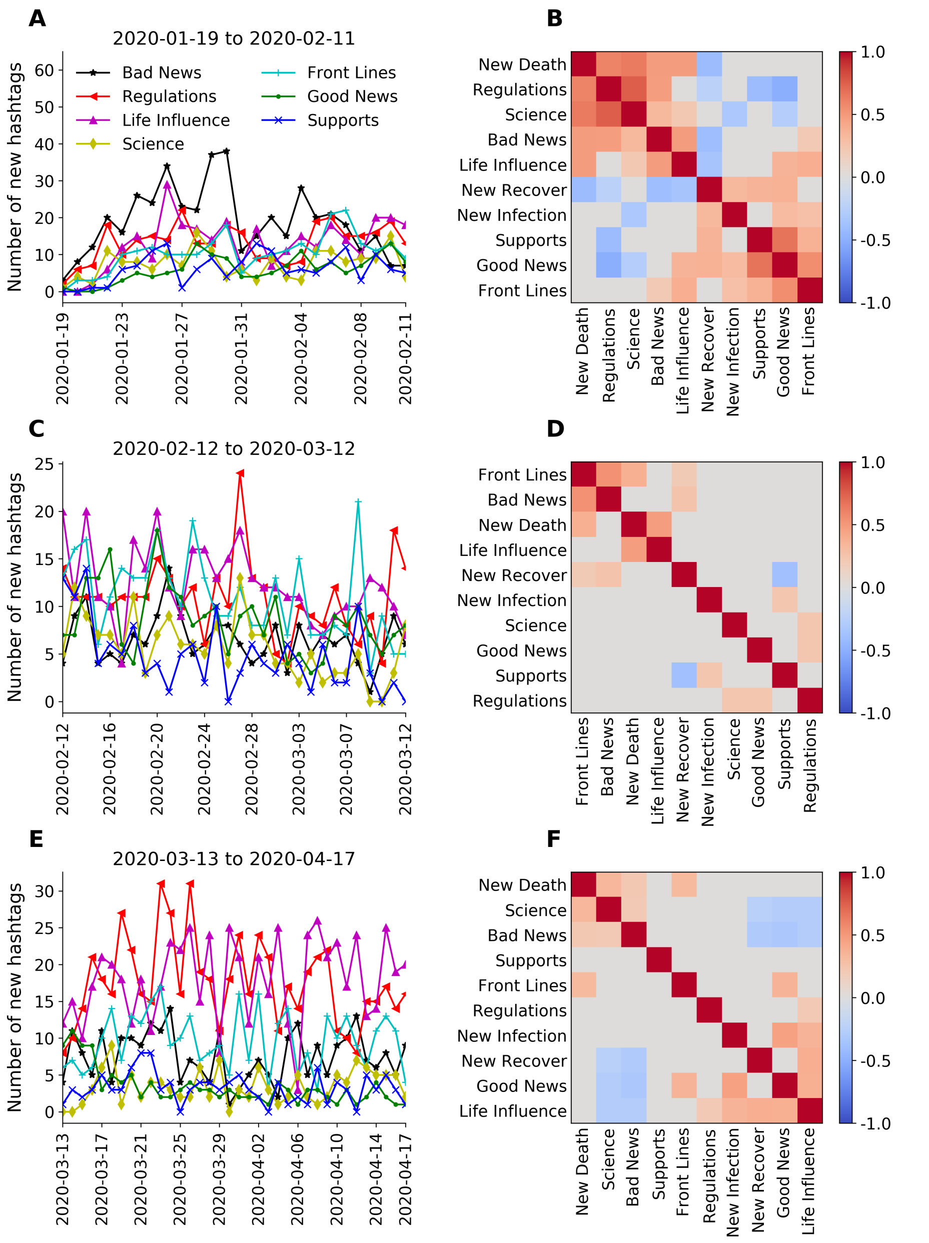

Figure SI2 Time series of daily new hashtags (unsmoothened) from the sub-categories of Mainland China COVID-hashtags and their correlation matrices with daily new infections, deaths, and recoveries, in the three periods after the outbreak. Correlations lower than 0.2 are considered as insignificant and are converted to zero.

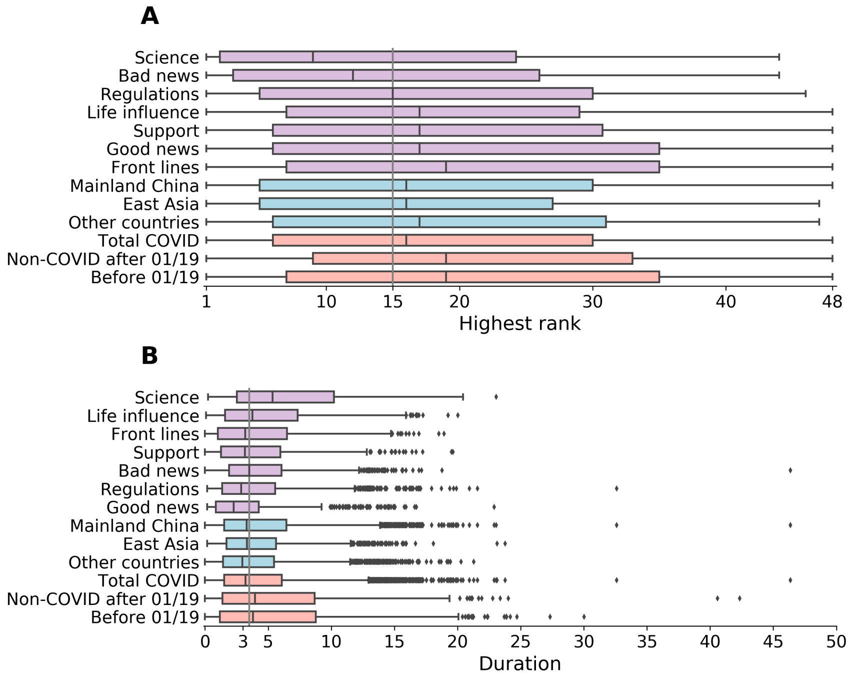

Figure SI3 Boxplots of the highest rank (A) and duration (B) of the different categories. In both plots, the purple categories are sub-categories of Mainland China category, which is colored in blue. The blue categories are sub-categories of Total COVID category, which is colored in orange. The dots in (B) are the outlier hashtags with long duration, e.g, the #疫情地图# (#Infection Map#) in the Bad New category and the #武汉再发现居家确诊病人将问责# (#(District Party Secretary) Will be accountable if home-confirmed patients found again in Wuhan#) in the Regulations category.

of normal and abnormal hashtag rank trajectory plots, and verify there are hashtags that stay at certain ranks such as rank 29 and 34 on the HSL for a strangely long time without any fluctuation.

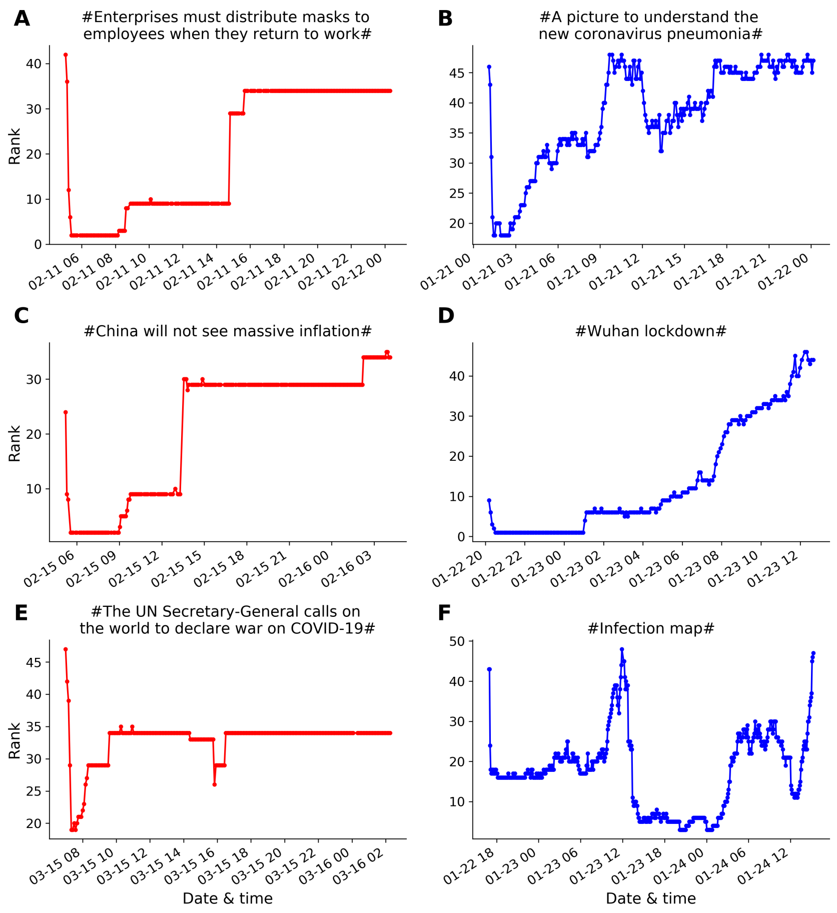

Figure SI4 Examples of rank trajectory plots of COVID-related hashtags. (A), (C), (E) Abnormal rank trajectory plots. (B), (D), (F) Normal rank trajectory plots.

Fig. SI4 shows examples of abnormal and normal rank trajectory plots of COVID-related hashtags on Weibo HSL. In Fig. SI4 (A), (C), (E), the ranks of the hashtags stay strangely long time at ranks 29 and 34, and then disappear from the HSL. Fig. SI4 (B), (D), (F) show relatively natural fluctuations in the rank trajectory plots. The example hashtags and their translations are shown in Table SI1. The abnormal rank plots are likely due to the algorithm intervention from Sina Weibo.

Table SI1 Chinese original and translations of example hashtags in Figure SI4.

Example Hashtags	Translation
#企业复工要为职工配发口罩#	#Enterprises must distribute masks to employees when they return to work#
#中国不会出现大规模通货膨胀#	#China will not see massive inflation#
#联合国秘书长呼吁全球共同向新冠宣战#	#The UN Secretary-General calls on the world to declare war on COVID-19#
#武汉封城#	#Wuhan lockdown#
#一图看懂新型冠状病毒肺炎#	#A picture to understand the new coronavirus pneumonia#
#疫情地图#	#Infection map#

References

1. Twitter Trend. https://trends24.in/about. Accessed August 8, 2020.
2. Zubiaga, A., Spina, D., Fresno, V., Martínez, R.: Classifying trending topics: A typology of conversation triggers on twitter. In: Berendt, B., de Vries, A., Fan, W. (eds.) Proceedings of the 20th ACM International Conference on Information and Knowledge Management, Glasgow, UK (2011). ACM, New York
3. Lee, K., Palsetia, D., Narayanan, R., Patwary, M.A., Agrawal, A., Choudhary, A.: Twitter trending topic classification. In: Spiliopoulou, M., et al. (eds.) Proceedings of the 11th IEEE International Conference on Data Mining Workshops, Vancouver, Canada, pp. 251–258 (2011). IEEE Computer Society, Los Alamitos
4. Tam, S., Hahm, D.: Exploring Coronavirus Twitter Trends. https://towardsdatascience.com/coronavirus-twitter-trends-d32fed5a027e. Accessed August 8, 2020. Towards Data Science, Inc. (2020)
5. COVID-19 stream. https://developer.twitter.com/en/docs/labs/covid19-stream/overview. Accessed August 7, 2020. Twitter, Inc. (2020)
